# Effects of Wnt5a overexpression in spinal cord injury

**DOI:** 10.1111/jcmm.16507

**Published:** 2021-05-03

**Authors:** Pau González, Carlos González‐Fernández, Francisco Javier Rodríguez

**Affiliations:** ^1^ Laboratory of Molecular Neurology Hospital Nacional de Parapléjicos Toledo Spain

**Keywords:** astrocyte, functional recovery, microglia, myelin preservation, neuron, NG2+ glial precursor, oligodendrocyte, serotonergic axons, spinal cord injury, Wnt5a

## Abstract

Accordingly to its known function in corticospinal tract (CST) developmental growth, previous reports have shown an inhibitory role of Wnt5a in CST regeneration after spinal cord injury (SCI). Interestingly, it has been subsequently demonstrated that Wnt5a also modulates the developmental growth of non‐CST axons and that different Wnt5a receptors are expressed in neurons, oligodendrocytes, NG2+ glial precursors and reactive microglia/macrophages and astrocytes after SCI. However, the role of Wnt5a in the response of these cell types, in the regeneration of non‐CST axons and in functional recovery after SCI is currently unknown. To evaluate this, rats were subjected to spinal cord contusion and injected with a lentiviral vector generated to overexpress Wnt5a. Histological analyses were performed in spinal cord sections processed for the visualization of myelin, oligodendrocytes, neurons, microglia/macrophages, astrocytes, NG2+ glial precursors and serotonergic axons. Motor and bladder function recovery were also assessed. Further advancing our knowledge on the role of Wnt5a in SCI, we found that, besides its previously reported functions, Wnt5a overexpression elicits a reduction on neuronal cell density, the accumulation of NG2+ glial precursors and the descending serotonergic innervation in the affected areas, along with impairment of motor and bladder function recovery after SCI.

## 1 INTRODUCTION

During the last decades, a great effort has been done to achieve a better understanding on the cellular and molecular processes that determine the progression and outcome of the damage after spinal cord injury (SCI) and, ultimately, unravel new potential therapeutic targets. Unfortunately, despite the advance in our knowledge about its pathophysiology, current clinically available therapeutic options only provide a very limited improvement in the histopathological and functional sequelae of SCI and, therefore, in its associated psychological, social and economic burden.^1^ In this regard, the Wnt family of proteins has gained the scientific community attention due to the mounting experimental evidence pointing to its relevance in the modulation of different essential processes that take place in this neuropathological condition.^2^, ^3^, ^4^, ^5^, ^6^, ^7^, ^8^, ^9^, ^10^, ^11^, ^12^, ^13^, ^14^, ^15^, ^16^, ^17^, ^18^ However, the potential involvement of the Wnt family of proteins in SCI is still far away from being well understood.

In mammals, the Wnt family of proteins is composed by nineteen Wnt ligands, ten conventional Frizzled (Fz) receptors, four non‐conventional receptors, two co‐receptors and fourteen soluble modulators. Depending on the combination of its different components, this complex family of proteins exerts its multiple functions by modulating at least three different signalling pathways: the canonical Wnt/β‐catenin and the non‐canonical Wnt/Ca^2+^ and Wnt/planar cell polarity (PCP) signalling pathways.^19^ Among the different Wnt ligands, several studies have pointed to a role of Wnt5a in SCI. More specifically, it has been shown that the expression of this Wnt ligand is significantly increased in the injured spinal cord.^2^, ^8^, ^16^ Moreover and according to its role during central nervous system (CNS) development,^20^ two different studies have strongly suggested that Wnt5a acts as a repellent for corticospinal tract (CST) regeneration after SCI.^8^, ^16^ Interestingly, we have subsequently demonstrated that, after SCI, the expression of different Wnt5a receptors such as Ryk, protein tyrosine kinase 7 (PTK7) and Fz5 is not only observed in axons, but also in different cell types such as neurons, astroglial cells, oligodendrocytes, NG2+ cells and microglia/macrophages located in the affected areas.^3^, ^4^, ^7^ These striking observations strongly suggest that, besides its functions in CST regeneration/retraction, Wnt5a might be also involved in the essential responses to SCI elicited by these cell types, in accordance with different studies showing the involvement of this Wnt ligand at least in neurodegeneration, microglial and astroglial activation and neuroinflammation in vitro and in vivo in other CNS pathologies.^11^, ^32^ Based on the previously detailed observations and to further elucidate the potential functions exerted by Wnt5a in the lesioned spinal cord, we aimed to evaluate the effects induced by lentiviral‐mediated Wnt5a overexpression in myelin preservation, neuronal and oligodendroglial cell survival, microglial and astroglial reactivity and NG2+ cell response in a clinically relevant model of rat SCI. In addition, we also sought to determine whether the previously detailed involvement of Wnt5a in SCI‐related CST regeneration/retraction might be reflected in other axonal tracts, by determining the effects induced by Wnt5a overexpression in the descending serotonergic innervation, which is also influenced by this Wnt ligand during development^33^, ^34^ and is particularly affected in this neuropathological condition.^35^ Finally, the potential induction of changes in functional recovery elicited by Wnt5a overexpression after SCI was also conveniently assessed.

## 2 MATERIALS AND METHODS

### 2.1 Production and titration of lentiviral vectors

Production and titration of self‐inactivating lentivirus were performed as already reported.^6^ Two different lentiviral vectors were generated, one that induce the expression of the green fluorescent protein (GFP) (lv‐GFP) and another that induce the expression of both Wnt5a and GFP (lv‐Wnt5a). To this end, the Wnt5a nucleotide sequence (NM_009524) tagged with hemagglutinin (HA) was cloned into the pWPI transfer plasmid (12 254, Addgene) (GenScript Biotech Corporation). A single batch of each lentiviral vector was used throughout the study.

### 2.2 Analysis of lv‐Wnt5a bioactivity

#### 2.2.1 Cell culture and transduction

All experiments carried out to analyse lv‐Wnt5a bioactivity were performed in B1a rat fibroblasts, which were cultured and transduced as we have previously described.^6^

#### 2.2.2 Quantitative Real‐Time PCR (qRT‐PCR)

SYBR Green‐based qRT‐PCR was used to evaluate whether lv‐Wnt5a was able to properly induce the mRNA expression of Wnt5a in B1a cells. Sample processing and amplification of specific transcripts were performed as previously detailed.^6^ Amplification of Wnt5a and 18s was carried out using 50 ng of complementary DNA and specific primers designed with the Primer Express software (Wnt5a: forward 5′‐AATAACCCTGTTCAGATGTCA‐3′, reverse 5′‐TACTGCATGTGGTCCTGATA‐3′; 18s: forward 5′‐ CGGCTACCACATCCAAGGAA‐3′, reverse 5′‐ GCTGGAATTACCGCGGCT‐3′). All gene expression analyses were performed in duplicate for each sample. Cycle threshold (Ct) values above 35 were considered as undetectable.

#### 2.2.3 Western blot

To determine whether lv‐Wnt5a‐induced Wnt5a protein expression in B1a cells and to evaluate the bioactivity of overexpressed Wnt5a at the Wnt signalling level, Western blot was used to quantify the protein levels of HA‐tagged Wnt5a, active β‐catenin, phosphorylated stress‐activated protein kinase/c‐Jun N‐terminal kinase (p‐SAPK/JNK) and phosphorylated Ca^2+^/calmodulin‐dependent protein kinase II (p‐CAMKII) in cell lysates as we have previously described.^6^ Western blot was also used to analyse the presence of overexpressed HA‐tagged Wnt5a in the culture medium as already reported.^6^ The different antibodies used for can be found in Table S1.

#### 2.2.4 Preparation of conditioned medium

Conditioned medium was obtained, as previously described,^6^ to determine the appropriate secretion of overexpressed Wnt5a to the extracellular space and to evaluate its biological activity at the Wnt signalling level.

### 2.3 Animals, surgical procedures and experimental design

To perform the present study, a total of 50 adult female Wistar rats were used (3 months; ≃ 250 g). Animal housing and experimental procedures were carried out in accordance with the Spanish (Royal Decree 53/2013) and the European Union (2010/63/EU) directives, and they were approved by the Bioethics Committee at The National Hospital of Paraplegics (Toledo, Spain) (Permit numbers 51/2009 and 45/2008). Spinal cord contusions and subsequent post‐operative cares were performed as previously described.^2^, ^3^, ^4^, ^7^, ^36^ Lentiviral vectors were intraparenchymatically injected immediately after spinal cord contusion following the method that we have previously reported.^6^ All efforts were done during the whole experimental process to minimize animal suffering. Animals were divided into two experimental groups: the first one composed by lesioned rats injected with lv‐GFP (GFP group) and the second one composed by lesioned rats injected with lv‐Wnt5a (Wnt5a group). Animals were sacrificed at 7 (n = 5 per group), 14 (n = 5 per group) and 126 days post‐injury (dpi) (n = 10 per group).

### 2.4 Histology

#### 2.4.1 Tissue processing

Anesthetized rats were sacrificed by intra‐aortic perfusion with 1 mg/kg of 4% paraformaldehyde (P6148, Sigma Aldrich). A 2 cm‐length spinal cord fragment containing the lesion was extracted, post‐fixed, cryoprotected, frozen, sectioned and stored as already reported.^2^, ^3^, ^4^, ^7^, ^36^

#### 2.4.2 Evaluation of transduction in vivo

The analysis of cell transduction after lentiviral injection in vivo was performed at 7, 14 and 126 dpi in a set of parallel spinal cord sections per animal processed as previously described.^6^

#### 2.4.3 Eriochrome cyanine staining

To evaluate the potential existence of changes in myelin preservation due to Wnt5a overexpression at 7, 14 and 126 dpi, eriochrome cyanine (ECy) staining was performed in a set of parallel spinal cord sections per animal, following the procedure detailed in previous reports.^6^, ^36^

#### 2.4.4 Immunohistochemistry

As previously detailed,^2^, ^3^, ^4^, ^6^, ^36^ we performed chromogen‐based simple immunohistochemistry in a set of parallel spinal cord sections per animal at 7, 14 and 126 dpi, for the visualization of glial fibrillary acidic protein (GFAP), ionized calcium‐binding adaptor molecule 1 (Iba1) and NG2 to evaluate the potential variations induced by Wnt5a overexpression in the presence of astrocytes, microglia macrophages and NG2+ cells, respectively. Fluorescence‐based simple immunohistochemistry was performed in a set of parallel spinal cord sections per animal at 126 dpi, for the visualization of neuronal nuclei (NeuN), adenomatous polyposis coli (APC) or serotonin (5‐HT) to determine the potential variations induced by Wnt5a overexpression in neuronal and oligodendroglial cell number, as well as in the location of 5‐HT axons, respectively. The experimental protocol used has been previously described.^6^, ^37^ The different antibodies used for both chromogen and fluorescence‐based immunohistochemical procedures can be found in Table S1. In all cases and to confirm a lack of undesired cross‐reactivity, spinal cord sections processed without the primary antibodies were used as controls. No non‐specific staining was observed in any case.

#### 2.4.5 Densitometric analysis

Image acquisition and densitometric analysis were carried out, as previously described,^6^, ^36^ in spinal cord sections processed for the visualization of myelin (ECy staining), astrocytes (GFAP immunostaining), microglia/macrophages (Iba1 immunostaining) and NG2+ cells (NG2 immunostaining) to evaluate the potential changes induced by Wnt5a overexpression in myelin preservation and in the presence of the previously detailed cell types after SCI. Densitometric analysis was also carried out to analyse the presence of 5‐HT axons in the lesioned spinal cord as we have detailed in a previous report.^6^

#### 2.4.6 Cell count

Following the methodology already described,^6^ automatized cell count in sections processed for the visualization of NeuN and APC was performed to determine the potential changes induced by Wnt5a overexpression in neuronal and oligodendroglial cell density, respectively.

### 2.5 Functional evaluation

#### 2.5.1 Open‐field test

The Basso, Beattie and Bresnahan (BBB) score and subscore for the open‐field test were carried out, as previously described,^6^, ^36^, ^38^ to determine the potential changes induced by Wnt5a overexpression in motor functional recovery after SCI. The BBB analysis was performed at 1, 3, 7, 14, 21, 28, 42, 56, 70, 84, 98, 112 and 126 dpi by two assessors who were blinded to the experimental groups.

#### 2.5.2 Catwalk gait analysis

As detailed in previous reports,^6^, ^36^, ^39^ motor functional recovery was also assessed using the CatWalk® gait analysis system (version 7.1, Noldus) either before (to obtain pre‐injury values) or after SCI (at 105 and 126 dpi). Only those lesioned animals displaying consistent stepping in the BBB open‐field test were included in the analysis (GFP group, n = 3, Wnt5a group, n = 4). The following gait parameters were analysed: regularity index, frequency of AB step patterns, hind paws base of support, print positions, hind paws stride length, hind paws duty cycle, hind paws swing duration, hind paws swing speed and hind paws stand duration.^39^

#### 2.5.3 Bladder function recovery

Among the different post‐operative cares performed, bladders were daily checked and emptied by manual expression until voiding control recovery (from 8:30am to 10:00AM). During this process, bladder size was evaluated and subdivided into four categories: large, medium, small and empty to identify the moment at which bladder function was recovered. Since non lesioned rats consistently displayed small (residual volume of urine during bladder expression) or empty (no urine during bladder expression) bladders, lesioned rats displaying small or empty bladder during three consecutive days were considered to have recovered bladder function.^40^, ^41^ No signs of urinary infections were observed.

### 2.6 Statistical analysis

Two‐way ANOVA followed by Bonferroni post hoc test was used to determine the existence of significant between‐group differences in data obtained from: (a) densitometrical analysis of sections processed for the visualization of myelin, microglia/macrophages, astrocytes, NG2+ glial precursors and 5HT+ axons, (b) neuronal and oligodendroglial cell count and iii) BBB open‐field test. One‐way ANOVA followed by Bonferroni post hoc test was used to determine the existence of significant between‐group differences in data obtained from: (a) qRT‐PCR‐based analysis of the mRNA expression of Wnt5a, (b) evaluation of the proper bioactivity of overexpressed Wnt5a at the Wnt signalling level by Western blot and (c) motor function assessment using the Catwalk gait analysis system. Two‐tailed Student's *t* test was used to determine the existence of significant between‐group differences in data obtained from the evaluation of the time to recover bladder function. In all cases, GraphPad Prism 5.01 software was used and a value of *P* ≤ .05 was considered statistically significant.

## 3 RESULTS

### 3.1 Analysis of lv‐Wnt5a bioactivity

To evaluate the proper function of lv‐Wnt5a, we firstly demonstrated that B1a cells transduced with lv‐Wnt5a (n = 3) displayed a robust increase in the mRNA expression of Wnt5a when compared to non‐transduced (NT) cells (n = 3) or transduced with lv‐GFP (n = 3) (Figure S1A). Likewise (Figure S1B), cell transduction with lv‐Wnt5a (n = 3) also evidently induced Wnt5a protein expression (Figure S1b_1_) (n = 3 per group). Moreover, we also found that overexpressed Wnt5a was also conveniently secreted to the extracellular medium, since HA‐tagged Wnt5a was detected in conditioned medium from cells transduced with lv‐Wnt5a (n = 3 per group) (Figure S1b_2_). We subsequently determined the effects induced by cell incubation during 1 hour with 100 or 200 ng/mL of recombinant Wnt5a (rWnt5a) (645‐WN/CF, RnD Systems) in the three currently best known Wnt‐related signalling pathways (Figure S1C). As shown, incubation of B1a cells with 200 ng/mL of rWnt5a did not modify the amount of active β‐catenin (Wnt/β‐catenin pathway) (Figure S1c_1_), decreased the presence of p‐CAMKII (Wnt/Ca^2+^ pathway) (Figure S1c_2_) and increased the levels of p‐SAPK/JNK (Wnt/PCP pathway) (Figure S1c_3_) (n = 3 per group). Finally, we found that in comparison with cell cultures incubated with conditioned medium (1:10) from cells transduced with lv‐GFP (CM lv‐GFP group, n = 3), cell cultures incubated during 1 hour with conditioned medium (1:10) from cell cultures transduced with lv‐Wnt5a (CM lv‐Wnt5a group, n = 3) induced similar modifications in these signalling pathways than those observed in cell cultures incubated during 1 hour with conditioned medium (1:10) from cell cultures transduced with lv‐GFP and supplemented with 200 ng/mL of rWnt5a [CM lv‐GFP +rWnt5a (200 ng/mL) group, n = 3] (Figure S1d_1‐3_).

### 3.2 Evaluation of transduction in vivo

Qualitative microscopic analysis of cell transduction at 7, 14 and 126 dpi showed that transduced GFP + cell distribution and amount were almost identical to that detailed in a previous report,^6^ where we used the same injection system. Succinctly, at all analysed times post‐injury GFP + cells were mainly found in rostral and caudal spinal cord levels neighbouring the lesion epicentre and, to a lesser extent, in the lesioned dorsal columns in rostro‐caudal spinal cord levels distant from the lesion epicentre. Finally, at 7 dpi GFP + cells were also found within the affected tissue and in the dorsal meninges at the lesion epicentre, although the presence of GFP + cells in this location was lower at 14 dpi and nearly absent at 126 dpi.

### 3.3 Wnt5a overexpression impairs descending serotonergic innervation after SCI

**FIGURE 1:**
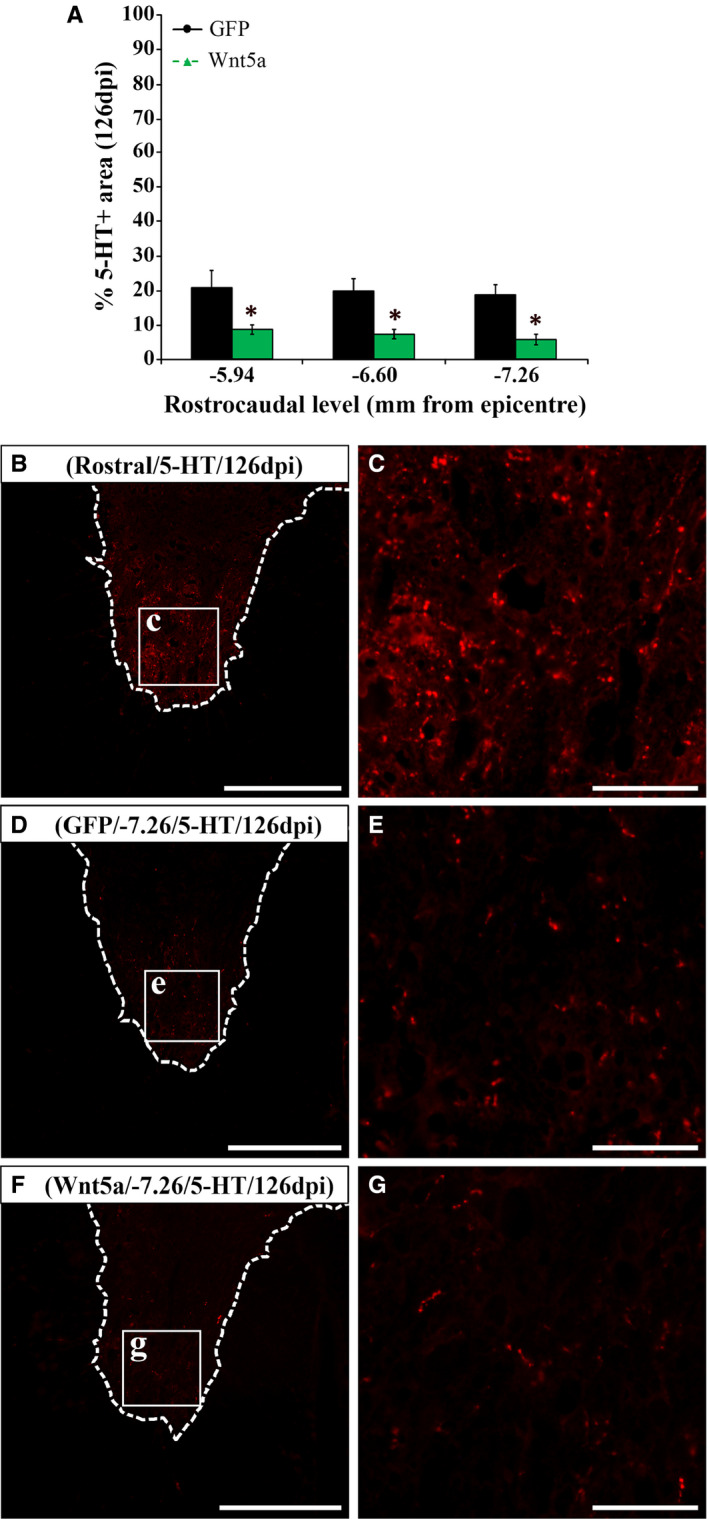
Wnt5a overexpression impairs descending motor serotonergic innervation after SCI. Figure showing data (A) and representative images (B‐G) obtained from the densitometric analysis of the serotonin (5‐HT) positive area in the ventral horns of spinal cord sections, corresponding to different rostro‐caudal levels (7.92 and 8.58 mm rostral and −5.94, 6.60 and 7.26 mm caudal from the lesion epicentre), from lesioned animals injected with a lentiviral vector that only induce the expression of GFP (GFP group, n = 10) or with a lentiviral vector that induce the expression of both GFP and Wnt5a (Wnt5a group, n = 10) at 126 days post‐injury (dpi). Squares in B, D and F indicate the areas shown in the corresponding higher magnification images. Scale bars B, D and F = 250 µm; scale bars in C, E and G = 40 µm. Data in A represent the percentage of 5‐HT + area in caudal spinal cord levels vs. that observed in rostral spinal cord levels and are presented as mean ± SEM. *P < .05 vs. GFP group

As previously introduced, we aimed to assess whether Wnt5a is able to modulate the regeneration of non‐CST axons after SCI. Since the serotonergic system plays a major role in the recovery of motor function, is particularly affected after SCI and is developmentally regulated by this Wnt ligand,^33^, ^34^, ^35^ we focused in the evaluation of the descending serotonergic innervation of the ventral horn motor region caudally to the injury site at 126 dpi. As shown (Figure 1A‐G), Wnt5a overexpression led to an evident reduction in the presence of 5‐HT + innervation in this region at the different evaluated caudal spinal cord levels.

### 3.4 Wnt5a overexpression reduces neuronal but not oligodendroglial cell density after SCI

**FIGURE 2:**
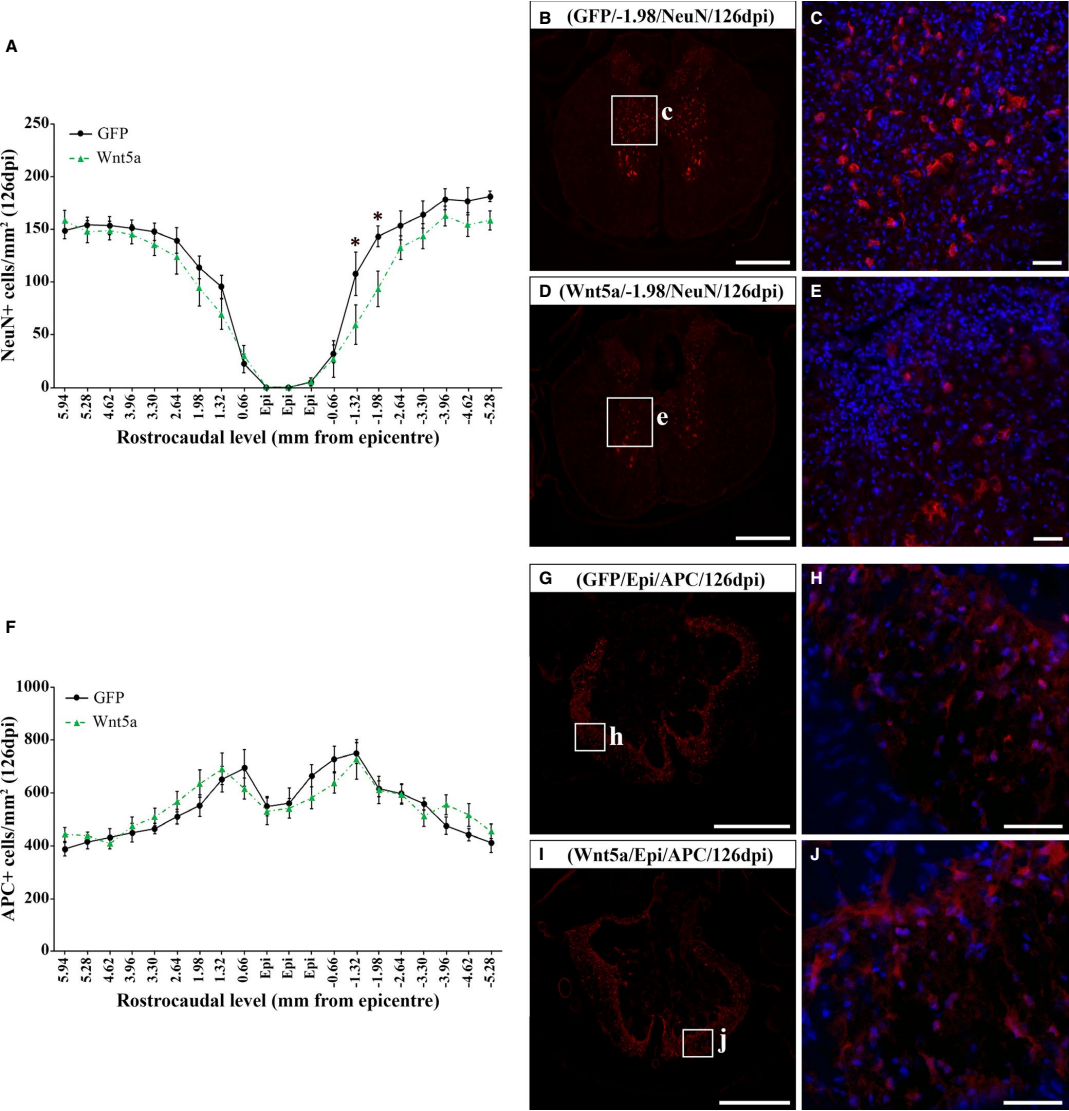
Wnt5a overexpression reduces neuronal cell density but does not affect oligodendroglial cell number after SCI. Figure showing data (A and F) and representative images (B‐E and G‐J) obtained from the quantification of neuronal (A‐E) and oligodendroglial (F‐J) cell density in spinal cord sections, corresponding to different rostro‐caudal levels, from lesioned animals injected with a lentiviral vector that only induce the expression of GFP (GFP group, n = 10) or with a lentiviral vector that induce the expression of both GFP and Wnt5a (Wnt5a group, n = 10) at 126 days post‐injury (dpi). For this purpose, sections were processed for the immunohistochemical visualization of neuronal nuclei (NeuN, neurons) or adenomatous polyposis coli (APC, oligodendrocytes). Squares in B, D, G and I indicate the areas shown in the corresponding higher magnification images. Scale bars in B, D, G and **i** = 500 µm; scale bars in C, E, H and J = 50 µm. Data are presented as mean ± SEM. *P < .05 vs. LV‐GFP group

Despite the different reports pointing to a role of Wnt5a in neurodegeneration,^21^, ^26^, ^29^, ^42^, ^43^ the potential involvement of Wnt5a in neuronal and oligodendroglial cell death after SCI is currently unknown. In this context, we found that Wnt5a overexpression led to a significant reduction in neuronal density at 126 dpi in caudal spinal cord levels adjacent to the lesion epicentre (Figure 2A‐E) where, noticeably, we found the higher accumulation of GFP+ transduced cells using this lentiviral injection method.^6^ No differences in oligodendroglial cell density between GFP and Wnt5a groups were found at the same time post‐injury (Figure 2F‐J).

### 3.5 Wnt5a overexpression does not influence myelin preservation after SCI

**FIGURE 3:**
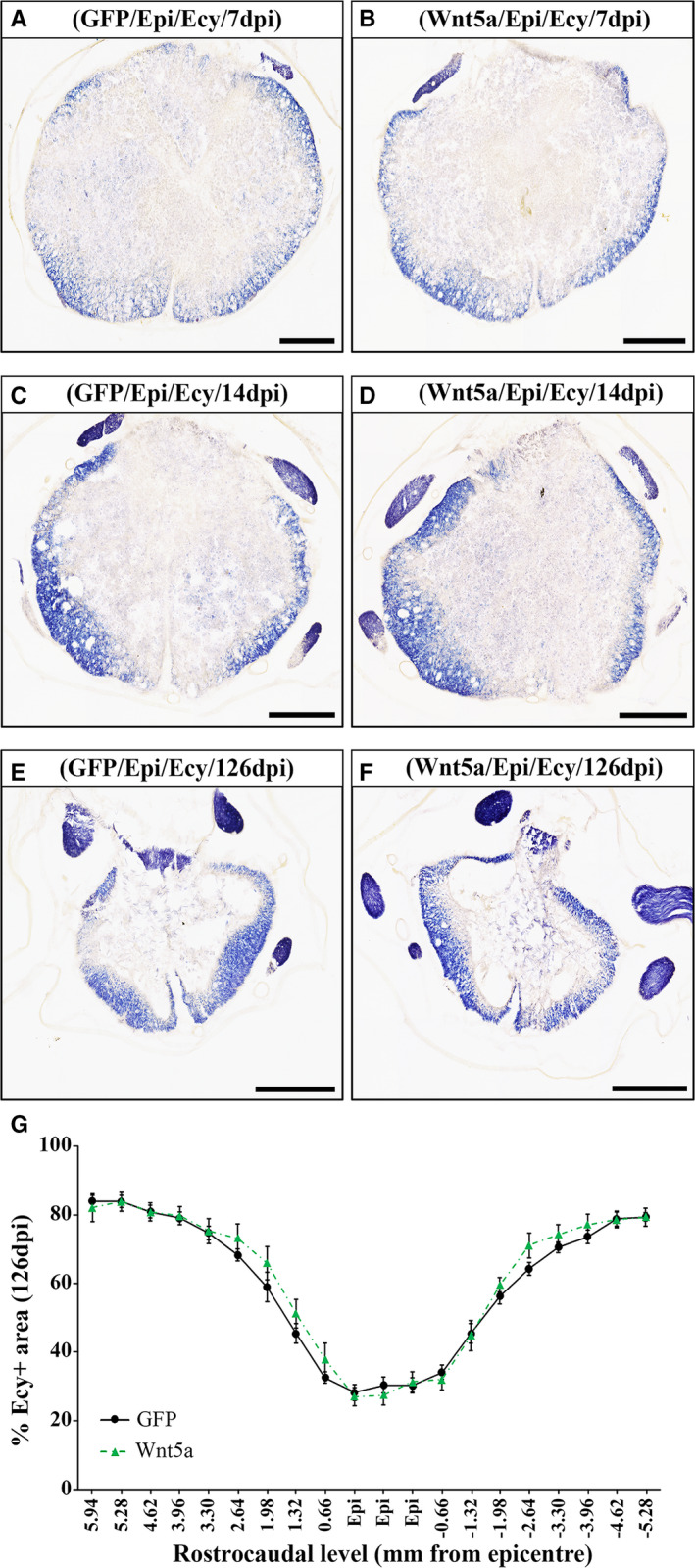
Wnt5a overexpression does not induce changes in myelin preservation after SCI. Figure showing representative images (A‐F) and data (G) obtained from the densitometric quantification of myelin preservation in spinal cord sections, processed for the visualization of eriochrome cyanine (ECy) and corresponding to different rostro‐caudal levels, from lesioned animals injected with a lentiviral vector that only induce the expression of GFP (GFP group) or with a lentiviral vector that induce the expression of both GFP and Wnt5a (Wnt5a group). Analysis was performed at 7 (A and B) (n = 5 per group), 14 (C and D) (n = 5 per group) and 126 (E‐G) (n = 10 per group) days post‐injury (dpi). Scale bars in A‐F = 500 µm. Data in G represent the percentage of ECy+area vs. total spinal cord area in each analysed rostro‐caudal level at 126 dpi and are presented as mean ± SEM. Data obtained from the evaluation of myelin preservation at 7 and 14 dpi can be found in Table S2.

To get further insight into the effects induced by Wnt5a overexpression after SCI, we next evaluated the existence of potential differences in myelin preservation, which is one of the most influencing aspects in the progression and outcome of SCI^44^ and is influenced by different Wnt components and/or signalling pathways.^6^, ^9^, ^14^ As shown, Wnt5a overexpression did not induce variations in the myelinated area at 7 (Figure 3A,B), 14 (Figure 3C,D) or 126 dpi (Figure 3E‐G) (Table S2 shows data obtained from the densitometric evaluation of myelin preservation at 7 and 14 dpi).

### 3.6 Wnt5a overexpression decreases NG2+ cell accumulation after SCI

**FIGURE 4:**
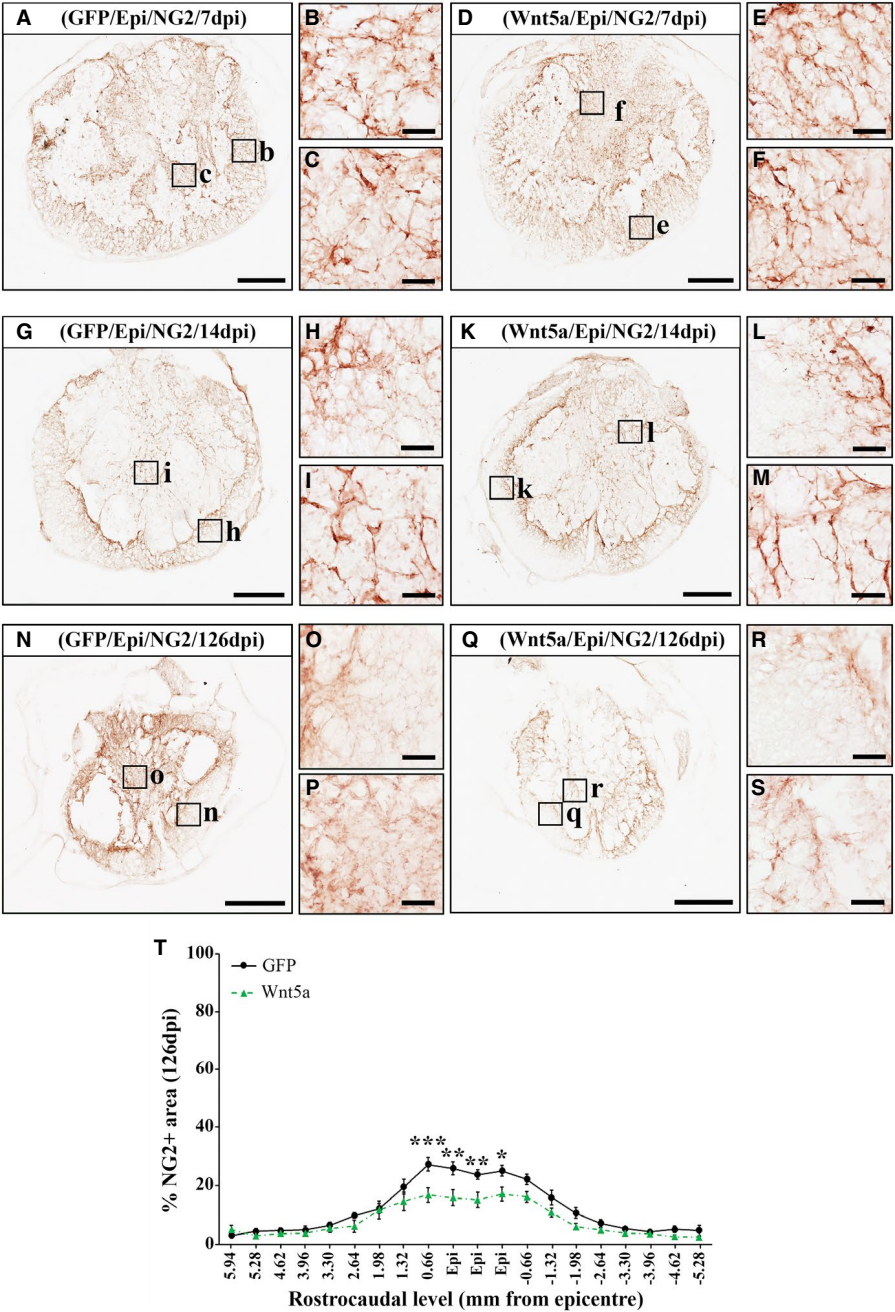
Wnt5a overexpression reduces NG2+ cell accumulation after SCI. Figure showing representative images (A‐R) and data (S) obtained from the densitometric quantification of the NG2 immunoreactive area in spinal cord sections, processed for the immunohistochemical visualization of NG2 and corresponding to different rostro‐caudal levels, from lesioned animals injected with a lentiviral vector that only induce the expression of GFP (GFP group) or with a lentiviral vector that induce the expression of both GFP and Wnt5a (Wnt5a group). Analysis was performed at 7 (A‐F) (n = 5 per group), 14 (G‐L) (n = 5 per group) and 126 (M‐S) (n = 10 per group) days post‐injury (dpi). Squares in A, D, G, J, M and P indicate the areas shown in the corresponding higher magnification images. Scale bars in A, D, G, J, M and P = 500 µm; scale bars in B, C, E, F, H, I, K, L, N, O, Q and R = 40 µm. Data in S represent the percentage of NG2+ area vs. total spinal cord area in each analysed rostro‐caudal level at 126 dpi and are presented as the mean ± SEM. *P < .05; **P < .01 and ***P < .001 vs. GFP group. Data obtained from the evaluation of the NG2+ cell response at 7 and 14 dpi can be found in Table S3

The response to SCI elicited by NG2+ glial precursors in the damaged tissue is also a major feature of SCI,^45^ which can be also modulated by specific components of the Wnt family of proteins and its related signalling pathways.^46^, ^47^, ^48^ Densitometric analysis of the NG2+ cell response showed that Wnt5a overexpression did not induce changes in the presence of NG2+ cells at 7 (Figure 4A‐F) and 14 dpi (Figure 4G‐L) (Table S3 shows data obtained from the densitometric evaluation of the NG2+ cell response at 7 and 14 dpi). However, when compared to that found in the GFP control group (Figure 4M‐O and S), animals from the Wnt5a group showed a significant reduction in the presence of NG2+ cells in rostro‐caudal spinal cord levels corresponding to the lesion epicentre at 126 dpi (Figure 4P‐S), which was evidently noted both in the lesion core (Figure 4O and R) and in the ring of spared tissue surrounding it (Figure 4N and Q).

### 3.7 Wnt5a overexpression does not modify astroglial and microglia/macrophage reactivity after SCI

**FIGURE 5:**
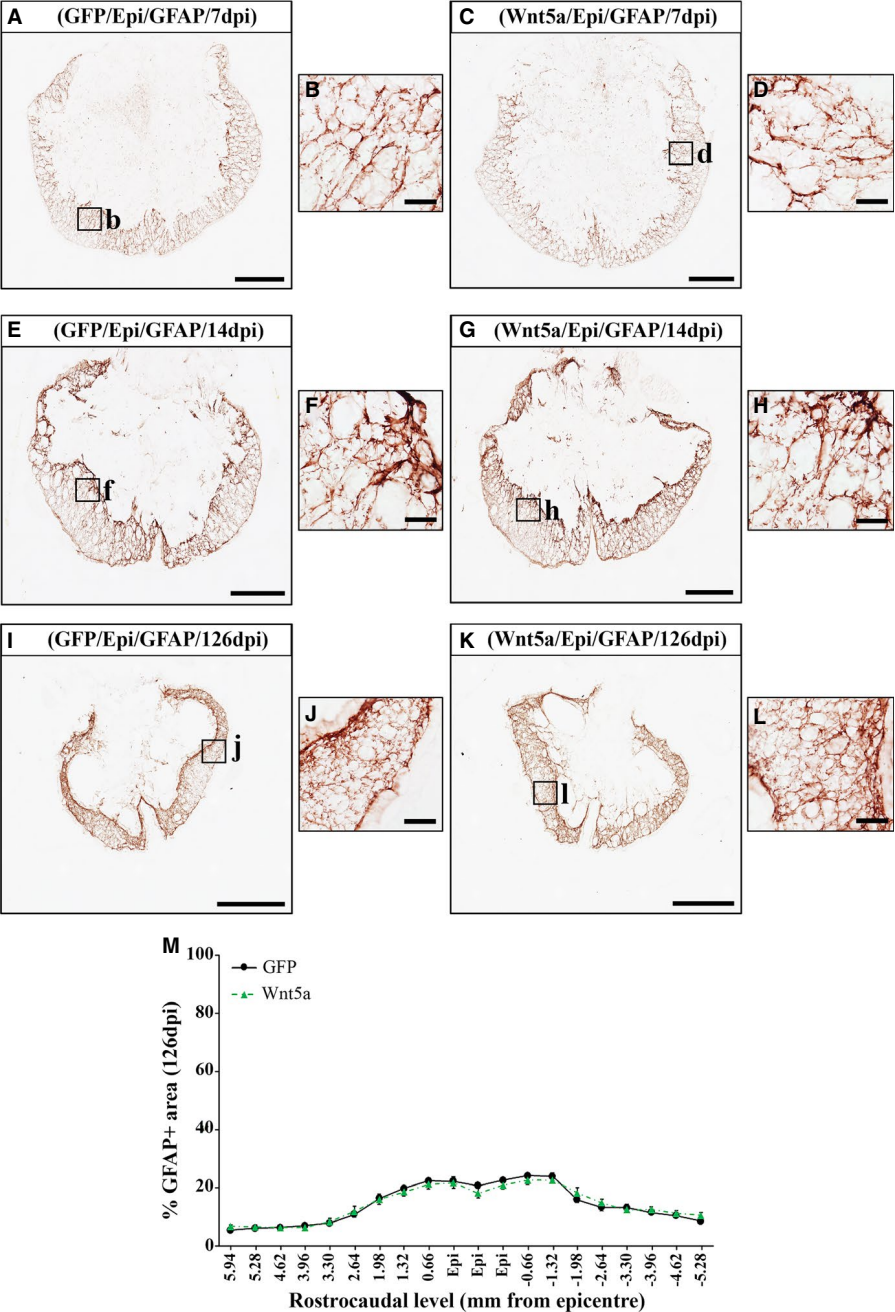
Wnt5a overexpression does not induce alterations in astroglial reactivity after SCI. Figure showing representative images (A‐L) and data (M) obtained from the densitometric quantification of the glial fibrillary acidic protein (GFAP) immunoreactive area in spinal cord sections, processed for the immunohistochemical visualization of GFAP and corresponding to different rostro‐caudal levels, from lesioned animals injected with a lentiviral vector that only induce the expression of GFP (GFP group) or with a lentiviral vector that induce the expression of both GFP and Wnt5a (Wnt5a group). Analysis was performed at 7 (A‐D) (n = 5 per group), 14 (E‐H) (n = 5 per group) and 126 (I‐L) (n = 10 per group) days post‐injury (dpi). Squares in A, C, E, G, I and K indicate the areas shown in the corresponding higher magnification images. Scale bars in A, C, E, G, I and **k** = 500 µm; scale bars in B, D, F, H, J and L = 40 µm. Data in M represent the percentage of GFAP+area vs. total spinal cord area in each analysed rostro‐caudal level at 126 dpi and are presented as mean ± SEM. Data obtained from the evaluation of the astroglial response at 7 and 14 dpi can be found in Table S4

**FIGURE 6:**
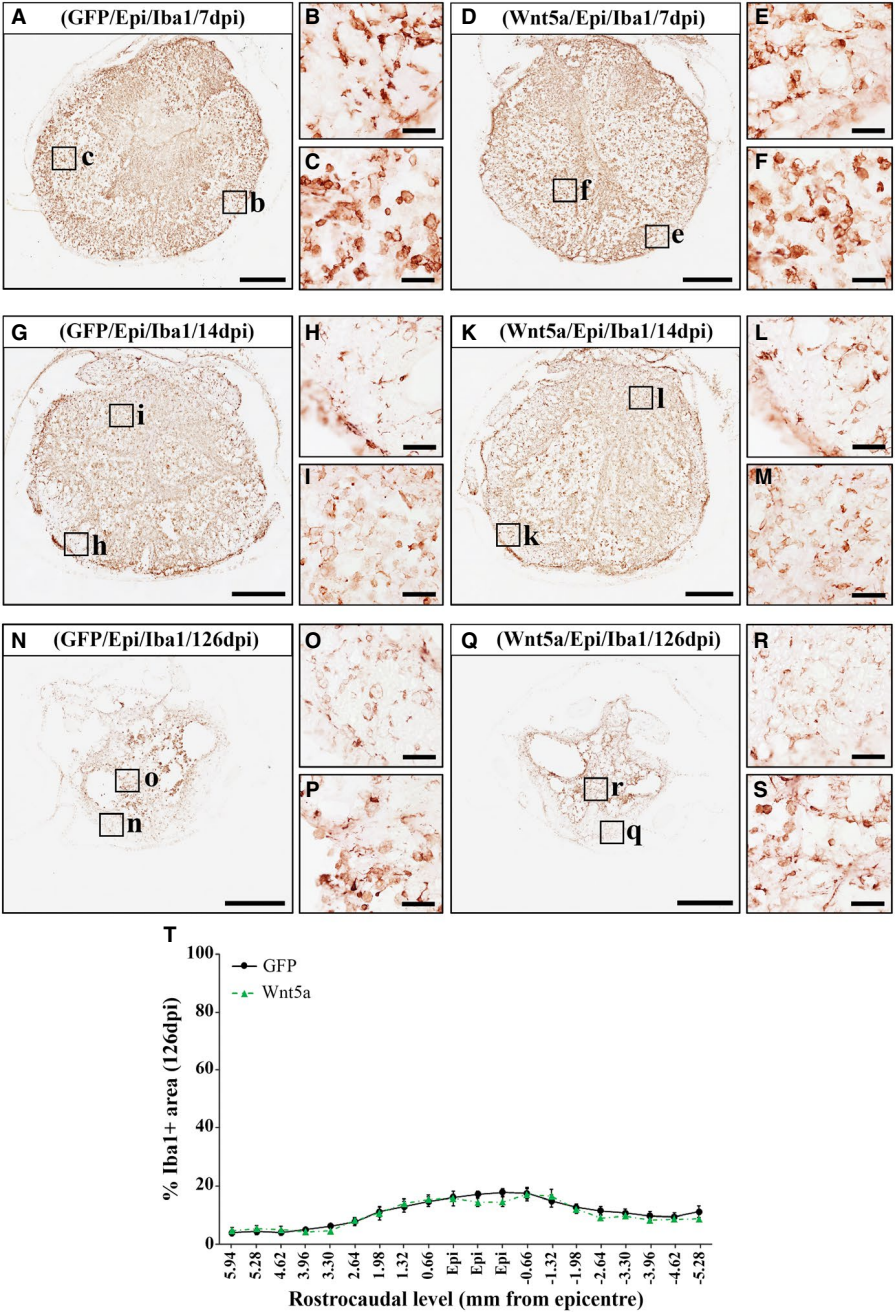
Wnt5a overexpression does not influence microglia/macrophage reactivity after SCI. Figure showing representative images (A‐R) and data (S) obtained from the densitometric quantification of the ionized calcium‐binding adaptor molecule 1 (Iba1) immunoreactive area in spinal cord sections, processed for the immunohistochemical visualization of Iba1 and corresponding to different rostro‐caudal levels, from lesioned animals injected with a lentiviral vector that only induce the expression of GFP (GFP group) or with a lentiviral vector that induce the expression of both GFP and Wnt5a (Wnt5a group). Analysis was performed at 7 (A‐F) (n = 5 per group), 14 (G‐L) (n = 5 per group) and 126 (M‐S) (n = 10 per group) days post‐injury (dpi). Squares in A, D, G, K, N and Q indicate the areas shown in the corresponding higher magnification images. Scale bars in A, D, G, J, M and P = 500 µm; scale bars in B, C, E, F, H, I, K, L, N, O, Q and R = 40 µm. Data in S represent the percentage of Iba1+ area vs. total spinal cord area in each analysed rostro‐caudal level at 126 dpi and are presented as mean ± SEM. Data obtained from the evaluation of the microglial response at 7 and 14 dpi can be found in Table S5

Given the upmost importance of the SCI‐related astroglial and microglial reactivity,^49^ together with the different reports pointing to the involvement of Wnt5a in glial activation and neuroinflammation,^11^, ^32^ we next aimed to assess whether Wnt5a overexpression modulates the astroglial and microglia/macrophage response after SCI. As shown, quantitative densitometric and qualitative microscopic analysis did not show differences, neither in the presence nor in the morphology of reactive astrocytes (Figure 5A‐M) and microglia/macrophages (Figure 6A‐S), in the affected areas at any of the different time points evaluated (7, 14 and 126 dpi) (data obtained from the densitometric evaluation at 7 and 14dpi of the astroglial and microglia/macrophage cell response can be found in Tables S4 and S5, respectively).

### 3.8 Wnt5a overexpression impairs functional recovery after SCI

**FIGURE 7:**
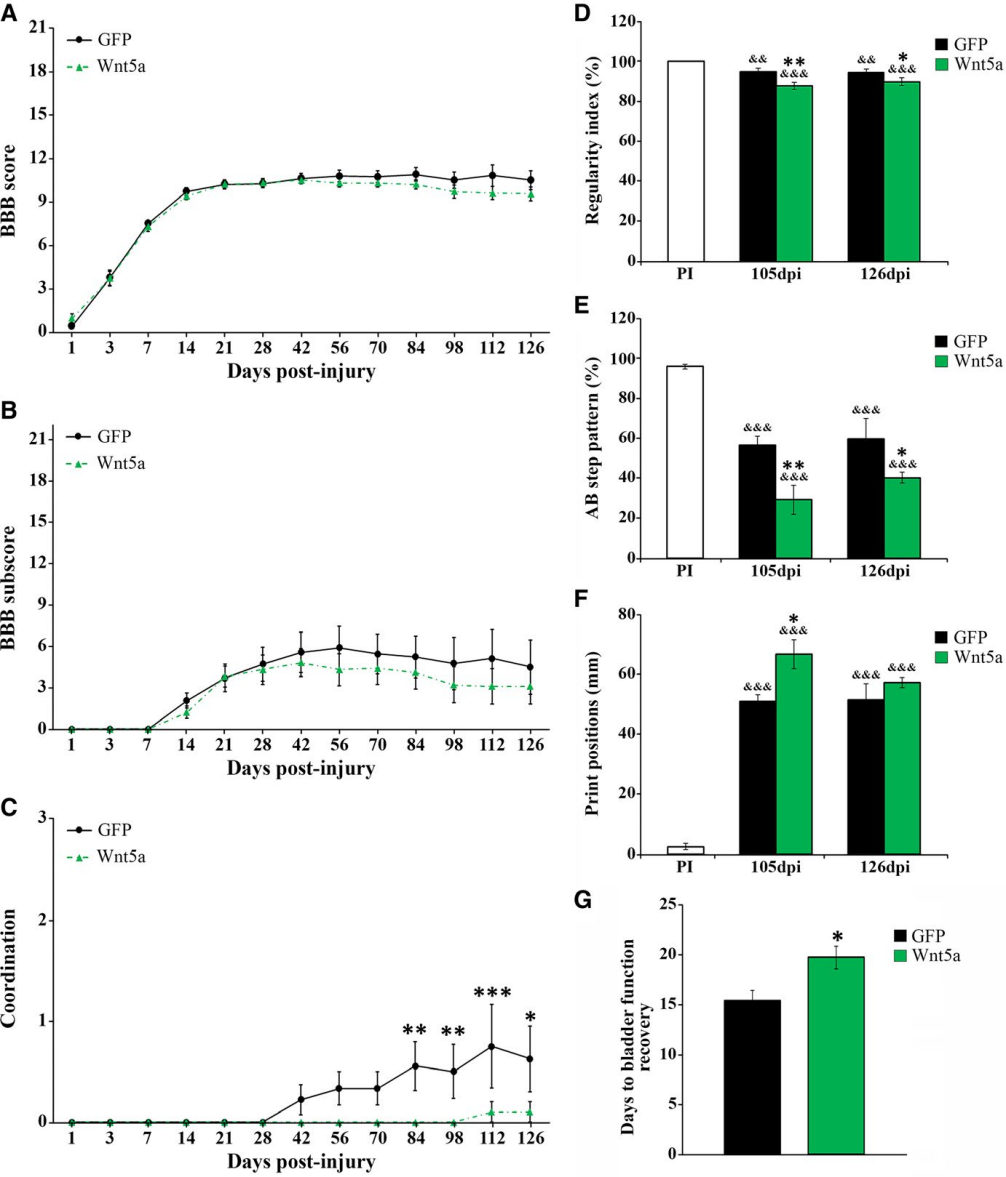
Wnt5a overexpression impairs functional recovery after SCI. Figure showing data obtained from the evaluation of motor functional recovery using the 21‐point BBB open‐field test (A, BBB score; B, BBB subscore; C, coordination) and the CatWalk® gait analysis system (D, regularity index; E, frequency of AB step pattern; F, print positions), as well as data obtained from the evaluation of bladder function recovery (G), in lesioned animals injected with a lentiviral vector that only induce the expression of GFP (GFP group) or with a lentiviral vector that induce the expression of both GFP and Wnt5a (Wnt5a group). The 21‐point point BBB open‐field test was carried out at 1, 3, 7, 14, 21, 28, 42, 56, 70, 84, 98, 112 and 126 days post‐injury (dpi). All animals used to evaluate the effects exerted by Wnt5a overexpression, which were sacrificed at 7 (n = 5 per group), 14 (n = 5 per group) and 126 (n = 10 per group) dpi, were included in the analysis. The evaluation of the different gait parameters using the CatWalk® gait analysis system was carried out either before injury, to obtain pre‐injury (PI) values, or after injury at both 105 and 126 dpi. Only those animals displaying consistent stepping were included in the analysis (GFP group, n = 3; Wnt5a group, n = 4). In all cases, data are presented as mean ± SEM. *, *P* <.05; **P < .01 and ***P, .001 vs. GFP group. &&, *P* < .01 and &&&, *P* < .001 vs. PI in D‐F. Data obtained from the analysis of the rest of the individual parameters evaluated using the 21‐point point BBB open‐field test and the CatWalk® gait analysis system can be found in Table [Link], [Link], respectively

Finally, although different reports have shown that the modulation of specific Wnt components and/or signalling pathways led to significant variations in functional recovery after SCI,^6^, ^18^ the potential involvement of Wnt5a in this essential issue has not been evaluated so far. We found that Wnt5a overexpression did not induce significant differences in the BBB score (Figure 7A) and subscore (Figure 7B). However, when the different individual aspects of locomotion analysed in the BBB open‐field test were evaluated separately, we found that Wnt5a overexpression led to a significant impairment in coordination (Figure 7C), which is a pivotal parameter of locomotion^50^, ^51^ (Table S6 shows data from the analysis of the rest of the individual BBB parameters evaluated). Accordingly, the analysis of motor function using the Catwalk gait analysis system showed that animals injected with lv‐Wnt5a displayed a significantly lower regularity index (Figure 7D) and percentage of AB step patterns (Figure 7E) at both 105 and 126 dpi. Moreover, Wnt5a overexpression also induced a significant increase in the print positions at 105 dpi (Figure 7F), while no significant between‐group differences were found in the rest of gait parameters evaluated (Table S7). Finally, a significant delay in the time to recover bladder function was observed in animals belonging to the Wnt5a group (Figure 7G).

## 4 DISCUSSION

In 2005, Liu and collaborators showed that Wnt5a is involved in the growth of CST axons during CNS development by acting as a repulsive cue through its interaction with the Ryk receptor.^20^ In agreement with the experimental evidence supporting that those molecular factors governing developmental axon pathfinding are also involved in axonal regeneration after injury,^52^ subsequent studies strongly suggested that the Wnt5a/Ryk signalling is also acting as a repulsive cue in the regeneration of the CST after SCI,^8^, ^16^ constituting the first and only demonstrations of the involvement of Wnt5a in SCI so far. Noticeably, it has been subsequently demonstrated that Wnt5a is also able to modulate the developmental growth of a wide range of types of non‐CST axons, including the serotonergic system.^33^, ^34^ However, its potential role in non‐CST axon regeneration after SCI is currently unknown. We show here that Wnt5a overexpression significantly reduced the serotonergic innervation of the ventral horn motor regions caudally to the injury site indicating that, in the lesioned spinal cord, Wnt5a does not only act as a repulsive factor for CST regeneration, but also for other axonal tracts that are critically involved in functional loss/recovery, such as the descending serotonergic system.^35^ Interestingly, this differs from what was observed during CNS development, when both descending and ascending serotonergic axons are attracted by Wnt5a via Fz3.^33^, ^34^ Given that the effects of Wnt5a in axonal growth are largely dictated by the available receptors in the axonal membrane,^53^ the differential expression of Wnt receptors in serotonergic axons during development or after injury might be underlying this apparent discrepancy.

Otherwise, recent reports performed by our group have opened a new avenue for Wnt5a research in SCI by demonstrating that, in the affected areas of the lesioned spinal cord, different Wnt5a receptors such as Fz5, Ryk and PTK7 are not only expressed at the axonal level, but also in neuronal somas, reactive astrocytes and microglia/macrophages, NG2+ glial precursors and oligodendrocytes.^3^, ^4^, ^7^ These observations indicate that these cell types are able to respond to Wnt5a and, thus, that this Wnt ligand might be influencing their critical responses to SCI. For instance, both Fz5 and Ryk are not only expressed in neurons in the healthy spinal cord but also in adjacent areas to the lesion epicentre after SCI,^3^, ^4^ which are thought to be affected by the secondary progression of the injury. Interestingly, previous reports have shown that Wnt5a modulates neuronal cell death/survival in cultured neurons under basal conditions^43^ or subjected to distinct noxious stimuli,^21^, ^29^, ^42^ as well as in vivo in animal models of chronic demyelination^26^ and cerebral ischemia.^29^ Although some apparent controversial observations can be found,^42^ most of these studies showed that Wnt5a increased neuronal death.^21^, ^26^, ^29^, ^43^ Accordingly, we found that Wnt5a overexpression reduced neuronal density in spinal cord levels adjacent to the lesion epicentre, supporting that Wnt5a is also a neurotoxic factor after SCI.

In addition, SCI also induces a prolonged death of oligodendroglial cells, leading to axonal demyelination and influencing the injury progression and outcome.^54^ Given the previously detailed involvement of Wnt5a in neuronal death/survival, together with prior reports showing that Wnt5a receptors such as Fz5 and Ryk are expressed in oligodendrocytes in the affected tissue after SCI,^3^, ^4^ we subsequently sought to evaluate whether Wnt5a might influence oligodendroglial cell survival in this neuropathological condition. As previously stated, we did not find differences in oligodendroglial cell density due to Wnt5a overexpression, supporting that this Wnt ligand does not affect oligodendroglial death/survival after SCI. Moreover, since oligodendrocytes are the myelinating cells in the CNS, we next analysed whether Wnt5a was able to modulate myelin preservation after SCI, as it has been previously described for other Wnt ligands such as Wnt1 and Wnt3a.^6^, ^18^ Accordingly to that observed in the oligodendroglial cell density, Wnt5a overexpression did not induce significant changes in the myelinated area. Furthermore, to obtain a general view of the functions exerted by Wnt5a in the oligodendroglial cell lineage after SCI, we also aimed to evaluate the potential changes induced by Wnt5a overexpression in the SCI‐related response of NG2+ glial precursors, which also express Wnt5a receptors such as Ryk, Fz5 and PTK7 in the damaged spinal cord tissue.^3^, ^4^, ^7^ Briefly, after SCI NG2+ glial precursors proliferate and migrate to the injury site where they can differentiate into either myelin‐producing oligodendrocytes or scar forming astrocytes, or accumulate in the glial scar, where they greatly influence glial scar formation and axonal regeneration.^45^ To our knowledge, only one previous study has attempted to evaluate the effects exerted by Wnt5a in NG2+ glial precursors,^11^ showing that lentiviral‐mediated Wnt5a overexpression in the healthy spinal cord did not influence the density and proliferation of this cell type, although slight qualitative morphological changes were observed. Interestingly, we found that Wnt5a overexpression significantly reduced the accumulation of NG2+ glial precursors in the lesion epicentre after SCI, suggesting that Wnt5a exerts different functions in this cell type in the spinal cord under physiological or pathological conditions, when they seem to be more responsive to this Wnt ligand. In this sense, we have recently shown that, while no expression of Ryk and PTK7 is found in NG2+ glial precursors in the healthy spinal cord, an evident expression of these Wnt5a receptors can be observed in this cell type after SCI, mainly in those NG2+ cells that are located in the lesion epicenter.^4^, ^7^ Finally, since the presence of oligodendroglial and astroglial cells was unaltered in the same time post‐injury and rostro‐caudal levels, it is plausible that the reduction observed in the accumulation of this cell type in the lesion epicentre might be due to a Wnt5a‐mediated decrease in its proliferation and/or survival, rather than an increase in their differentiation rate.

Another major hallmark of SCI is the injury‐associated activation of astroglial and microglial cells, which suffer evident morphological and functional alterations that allow them to exert both beneficial and deleterious functions that, in turn, greatly determine the progression and outcome of the injury.^55^ Interestingly and as previously introduced, different reports have pointed to a role of Wnt5a in astroglial and microglial activation under both physiological and pathological conditions,^11^, ^22^, ^23^, ^25^, ^27^, ^28^, ^31^ although its potential role in SCI‐related astroglial and microglia/macrophage reactivity has not been currently evaluated. Considering the previously detailed observations, together with the increased expression of different Wnt5a receptors observed in reactive microglia/macrophages and astrocytes in the affected areas after SCI,^3^, ^4^, ^7^ it appears surprising that Wnt5a overexpression did not induce changes in the presence of reactive microglia/macrophages and astroglial cells in the injured spinal cord. These observations suggest that this Wnt ligand does not affect the proliferation and/or recruitment of these cell types in this neuropathological condition and that, as previously suggested,^23^, ^25^ the function of Wnt5a on astroglial and microglial reactivity is finely tuned depending on the biological context. Further systematic studies will be needed to ascertain the specific mechanisms and circumstances underlying the differential responses elicited by Wnt5a in astrocytes and microglia.

Finally, we found that Wnt5a overexpression after SCI significantly hindered motor functional recovery mainly by impairing interlimb coordination, which is a major feature of locomotion that is severely affected after SCI in both quadrupedals and bipedals, and represents an important outcome measure of locomotor performance.^50^, ^51^ Moreover, SCI not only results in motor deficits but also in severe autonomic disabilities.^56^ Among them, one of the most critical consequences of SCI is bladder dysfunction,^57^ which is a principal concern of SCI patients since it is a considerable threat for their quality of life.^58^ In rodents, SCI initially results in a period of bladder areflexia characterized by the presence of large residual urine volumes that cause bladder overdistension, which is usually resolved after approximately the first two weeks after SCI, when the lesioned animals recover voiding control.^59^ In this regard, we found that the overexpression of Wnt5a also significantly delayed bladder function recovery after SCI. Although we cannot conclude the specific cellular and/or molecular mechanisms underlying the impairment in functional recovery observed due to Wnt5a overexpression, it should be noted that different reports have shown a consistent relationship between the preservation and function of the descending serotonergic system and the recovery of interlimb coordination and bladder function after SCI.^35^, ^60^

In conclusion, in the present study we show that Wnt5a in the lesioned spinal cord is more than a regulator of CST regeneration, since Wnt5a overexpression after SCI led to a reduction in the descending serotonin innervation and decreased neuronal cell density and NG2+ cell accumulation in the injured areas, without affecting the density of oligodendroglial cells, myelin preservation and the presence of reactive astroglial and microglial cells. More importantly, we also demonstrate that Wnt5a overexpression significantly impaired functional recovery after SCI. Finally, since we and others have previously found that the endogenous expression of Wnt5a is significantly increased in the injured spinal cord,^2^, ^8^ the results obtained suggest that the inhibition or blockade of endogenous Wnt5a might be a potential therapeutic approach to ameliorate the SCI‐associated histopathological and functional deficits. Future studies will be needed to test this interesting hypothesis.

## CONFLICT OF INTEREST

The authors declare that no conflict of interest exists.

## AUTHOR CONTRIBUTIONS

**Pau González:** Conceptualization (equal); Data curation (equal); Formal analysis (equal); Investigation (equal); Methodology (equal); Supervision (equal); Validation (equal); Visualization (equal); Writing‐original draft (equal); Writing‐review & editing (equal). **Carlos González‐Fernández:** Conceptualization (supporting); Data curation (equal); Formal analysis (equal); Investigation (equal); Methodology (equal); Writing‐review & editing (equal). **Francisco Javier Rodriguez:** Conceptualization (equal); Funding acquisition (equal); Investigation (equal); Methodology (equal); Project administration (equal); Resources (equal); Supervision (equal); Validation (equal); Writing‐review & editing (equal).

## Supporting information

Figure S1

Table S1

Table S2

Table S3

Table S4

Table S5

Table S6

Table S7

## Data Availability

The data that support the findings of this study are available from the corresponding author upon reasonable request.
