# Supplementary material for: Effects of Wnt5a overexpression in spinal cord injury
Source: J Cell Mol Med. 2021 May 3;25(11):5150–63. doi: 10.1111/jcmm.16507 (PMC8178287; doi:10.1111/jcmm.16507)
Supplement: Supplementary file 1 — Figure S1 [file JCMM-25-5150-s003.pdf]

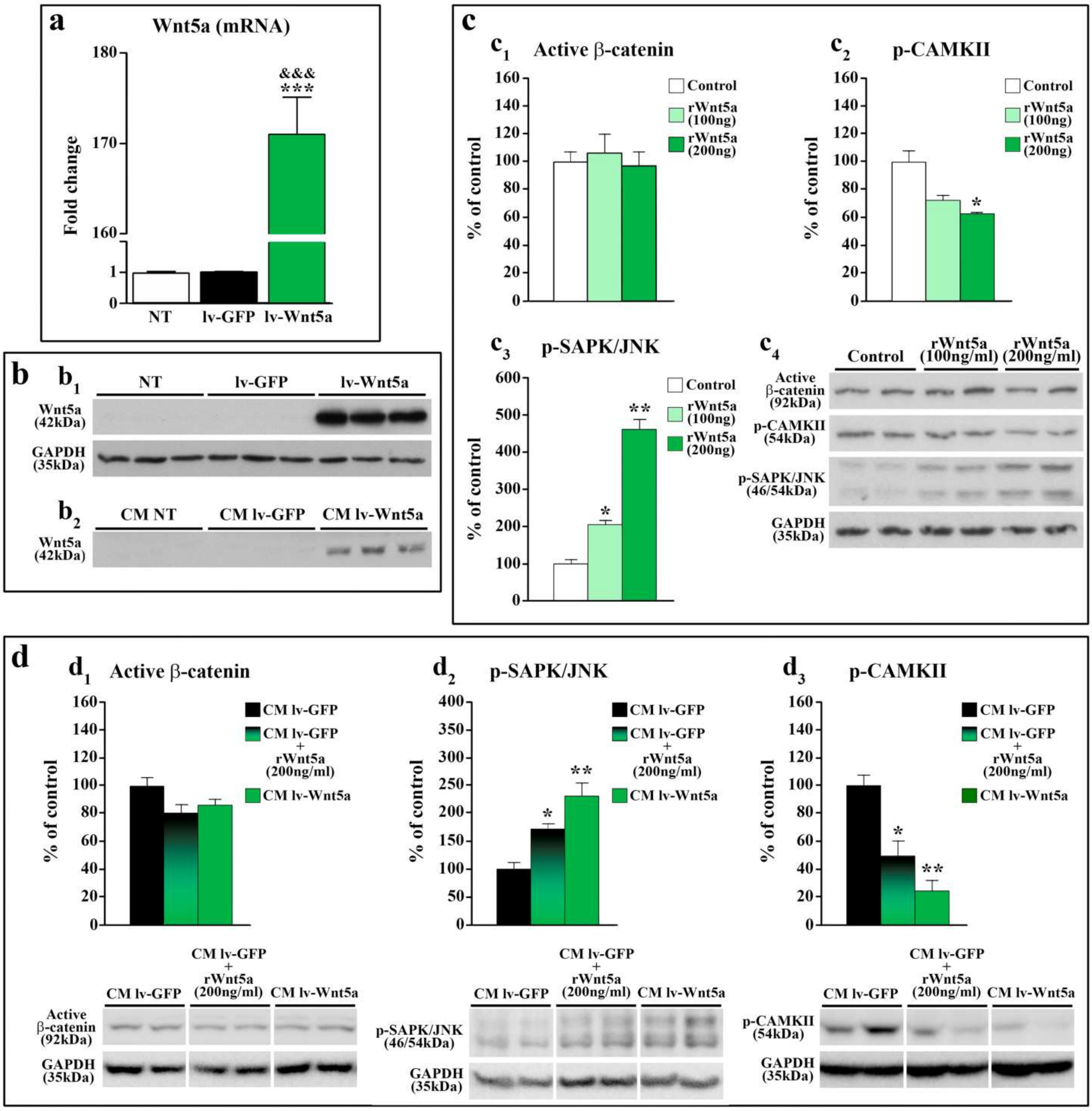

Figure S1. Evaluation of the lentiviral vector generated to overexpress Wnt5a (lv-Wnt5a) functioning. Figure showing data obtained from the different experiments performed in cultured B1a cells to evaluate lv-Wnt5a functioning. **a**, evaluation of Wnt5a mRNA expression in non-transduced (NT group, n = 3) cells and transduced with lv-GFP (lv-GFP group, n = 3) or lv-Wnt5a (lv-Wnt5a group, n = 3) (***, $p < 0.001$ vs. NT; &&&, $p < 0.001$ vs. GFP). **b**, representative Western blot bands obtained from the analysis of the presence of overexpressed HA-tagged Wnt5a in cell lysates (NT group, n = 3; GFP group, n = 3; Wnt5a group, n = 3) (**b$_1$**) and conditioned medium (CM) (**b$_2$**) from NT (CM NT group, n = 3), lv-GFP (CM lv-GFP group, n = 3) and lv-Wnt5a (CM lv-Wnt5a group, n = 3) cells. **c**, data obtained from the Western blot-based analysis of the effects exerted by the incubation of B1a cells during 1 h with

100 or 200 ng/ml ($n = 3$ per group) of recombinant Wnt5a (rWnt5a) in the protein levels of active β-catenin (**c_1**), phosphorylated Ca^{2+}/calmodulin-dependent protein kinase II (p-CAMKII) (**c_2**) and phosphorylated stress-activated protein kinase/c-Jun N-terminal kinase (p-SAPK/JNK) (**c_3**) (representative Western blot bands are shown in **c_4**) (*, $p < 0.05$ and **, $p < 0.01$ vs. non-treated control cells). **d**, data and representative bands obtained from the Western blot-based evaluation of the effects induced by the incubation of B1a cells with CM (1:10) from lv-GFP cells (CM lv-GFP group, $n = 3$), lv-GFP cells supplemented with 200 ng/ml of rWnt5a [CM lv-GFP + rWnt5a (200 ng/ml) group, $n = 3$] or lv-Wnt5a (CM lv-Wnt5a group, $n = 3$) cells in the protein levels of active β-catenin (**d_1**), p-CAMKII (**d_2**) and p-SAPK/JNK (**d_3**) (*, $p < 0.05$ and **, $p < 0.01$ vs. CM lv-GFP group). In all cases, data are presented as mean ± SEM.
