# Supplementary material for: Effects of Wnt5a overexpression in spinal cord injury
Source: J Cell Mol Med. 2021 May 3;25(11):5150–63. doi: 10.1111/jcmm.16507 (PMC8178287; doi:10.1111/jcmm.16507)
Supplement: Supplementary file 2 — Table S1 [file JCMM-25-5150-s002.pdf]

**Experimental Technique**	**Antibody**	**Supplier and reference**	**Dilution**
Western blot	Rabbit polyclonal anti-HA	Sigma Aldrich, H6908	1:5000
	Mouse monoclonal anti-active β-catenin	Millipore, 05-665	1:500
	Mouse monoclonal anti-p-SAPK/JNK	Cell Signaling, #9255	1:1000
	Mouse monoclonal anti-p-CAMKII	Abcam, ab171095	1:500
	Mouse monoclonal anti-GAPDH	Abcam, ab8245	1:10000
	HRP-linked goat anti-mouse	Thermo Fisher, 31430	1:7000
	HRP-linked goat anti-rabbit	Thermo Fisher, 31460	1:5000
Chromogen-based immunohistochemistry	Mouse monoclonal anti-GFAP	Sigma Aldrich, G3893	1:1000
	Rabbit polyclonal anti-Iba1	Wako, 019-19741	1:1000
	Rabbit polyclonal anti-NG2	Millipore, AB5320	1:250
	Biotinylated horse anti-mouse	Vector, BA-2001	1:500
	Biotinylated goat anti-rabbit	Vector, BA-1000	1:500
Fluorescence-based immunohistochemistry	Mouse monoclonal anti-APC	Calbiochem, OP80	1:100
	Rabbit monoclonal anti-NeuN	Millipore, MABN140	1:100
	Rabbit polyclonal anti-5-HT	Sigma Aldrich, S5545	1:500
	Dylight594-linked goat anti-rabbit	Abcam, ab96897	1:500
	Dylight594-linked goat anti-mouse	Abcam, ab96881	1:500

Table S1. Table showing the primary and secondary antibodies used to perform the different Western blot and immunohistochemical-based analysis. HA, hemagglutinin; p-SAPK/JNK, phosphorylated stress-activated protein kinase/c-Jun N-terminal kinase; p-CAMKII, phosphorylated Ca^{2+}/calmodulin-dependent protein kinase II; GAPDH, glyceraldehyde 3-phosphate dehydrogenase; HRP, horseradish peroxidase; GFAP, glial fibrillary acidic protein; Iba1, ionized calcium binding adaptor molecule 1; APC, adenomatous polyposis coli; NeuN, neuronal nuclei; 5-HT, serotonin.
