# Supplementary material for: Effects of Wnt5a overexpression in spinal cord injury
Source: J Cell Mol Med. 2021 May 3;25(11):5150–63. doi: 10.1111/jcmm.16507 (PMC8178287; doi:10.1111/jcmm.16507)
Supplement: Supplementary file 3 — Table S2 [file JCMM-25-5150-s004.pdf]

		Rostro-caudal levels (mm from epicenter)																				
		**5.94**	**5.28**	**4.62**	**3.96**	**3.30**	**2.64**	**1.98**	**1.32**	**0.66**	**Epi**	**Epi**	**-0.66**	**-1.32**	**-1.98**	**-2.64**	**-3.30**	**-3.96**	**-4.62**	**-5.28**	**-5.94**	
**GFP**	**Mean**	88.4	89.1	82.4	87.2	85.1	81.2	75.3	63.6	41.1	10.0	8.4	31.1	55.5	63.9	70.6	76.8	79.4	82.1	81.1	81.7	**7 dpi**
	**SEM**	2.5	1.1	0.9	2.6	3.8	3.3	5.7	7.0	6.6	1.2	1.1	5.6	2.9	1.9	3.1	2.1	1.2	2.3	3.3	2.3	
**Wnt5a**	**Mean**	87.1	86.3	86.6	86.6	80.1	75.4	72.6	57.6	35.1	10.9	7.9	18.9	44.8	59.8	62.5	69.4	75.2	76.9	79.8	7.0	
	**SEM**	1.0	1.5	1.3	1.1	1.0	2.1	2.8	5.9	7.1	2.5	1.5	3.6	3.8	1.9	2.3	2.7	1.5	3.1	2.2	2.2	
**GFP**	**Mean**	89.3	87.0	86.8	85.4	83.3	82.1	82.8	63.3	31.5	13.0	14.1	35.0	60.8	71.8	73.9	80.5	82.6	83.6	83.8	87.8	**14 dpi**
	**SEM**	1.1	1.7	1.0	1.0	2.3	0.5	1.4	4.0	7.6	4.3	5.8	7.4	4.2	4.0	2.6	0.7	2.2	2.8	2.8	0.5	
**Wnt5a**	**Mean**	91.3	91.6	89.5	86.2	87.6	83.8	80.5	68.6	35.8	14.0	13.6	40.1	69.3	77.2	79.9	81.0	83.0	85.3	84.5	84.5	
	**SEM**	2.5	2.3	2.7	4.0	3.7	3.0	2.2	4.8	5.7	5.2	3.3	8.1	2.4	4.8	6.1	4.4	3.7	1.8	1.9	1.8	

Table S2. Table showing data obtained from the densitometric analysis of myelin preservation at 7 and 14 days post-injury (dpi). Please note that data obtained from the evaluation of this parameter at 126 dpi can be found in Figure 3. Data represent the percentage of Eriochrome cyanine (Ecy)+ area vs. total spinal cord area in each analyzed rostrocaudal level, and are presented as mean ± SEM. GFP group, lesioned animals injected with a lentiviral vector generated to overexpress GFP; Wnt5a group, lesioned animals injected with a lentiviral vector generated to overexpress both GFP and Wnt5a.
