# Supplementary material for: Effects of Wnt5a overexpression in spinal cord injury
Source: J Cell Mol Med. 2021 May 3;25(11):5150–63. doi: 10.1111/jcmm.16507 (PMC8178287; doi:10.1111/jcmm.16507)
Supplement: Supplementary file 4 — Table S3 [file JCMM-25-5150-s007.pdf]

		Rostro-caudal levels (mm from epicenter)																				
		**5.94**	**5.28**	**4.62**	**3.96**	**3.30**	**2.64**	**1.98**	**1.32**	**0.66**	**Epi**	**Epi**	**-0.66**	**-1.32**	**-1.98**	**-2.64**	**-3.30**	**-3.96**	**-4.62**	**-5.28**	**-5.94**	
**GFP**	**Mean**	3.5	2.9	3.1	4.0	6.7	9.5	11.7	15.8	22.9	26.9	24.5	25.0	24.5	16.2	17.7	13.1	9.1	11.4	10.5	10.6	**7dpi**
	**SEM**	1.2	0.5	0.6	0.8	0.6	2.2	3.0	2.3	1.3	1.8	1.7	1.5	2.2	1.0	1.3	1.8	1.3	1.9	2.2	2.9	
**Wnt5a**	**Mean**	4.6	4.6	3.9	3.0	5.3	7.0	9.0	17.7	19.9	23.1	22.3	21.8	20.5	15.1	13.4	9.3	7.8	7.4	7.1	9.7	
	**SEM**	1.6	1.6	0.9	0.7	1.4	0.6	1.5	1.3	1.0	1.2	0.8	1.4	1.4	2.1	1.9	0.5	1.4	0.6	1.3	2.1	
**GFP**	**Mean**	4.0	5.9	6.5	5.1	6.0	5.0	7.6	14.3	17.2	21.1	20.1	17.8	14.6	14.5	9.3	7.2	7.3	5.8	6.4	4.9	**14dpi**
	**SEM**	0.2	0.7	2.1	0.8	1.6	0.8	1.3	0.8	1.2	2.5	1.2	1.6	2.5	0.8	1.3	0.7	0.5	0.5	1.1	0.5	
**Wnt5a**	**Mean**	3.6	6.9	8.5	6.9	5.2	6.8	9.3	12.5	17.1	17.5	19.5	17.9	16.1	13.4	5.6	7.3	10.1	8.8	8.1	7.3	
	**SEM**	1.6	2.6	2.3	2.3	0.5	1.1	2.6	2.1	2.4	1.7	2.1	2.2	3.3	3.8	1.5	1.4	2.6	2.0	2.4	2.1	

Table S3. Table showing data obtained from the densitometric analysis of the NG2+ cell response at 7 and 14 days post-injury (dpi). Please note that data obtained from the evaluation of this parameter at 126 dpi can be found in Figure 4. Data represent the percentage of NG2+ area vs. total spinal cord area in each analyzed rostrocaudal level, and are presented as mean ± SEM. GFP group, lesioned animals injected with a lentiviral vector generated to overexpress GFP; Wnt5a group, lesioned animals injected with a lentiviral vector generated to overexpress both GFP and Wnt5a.
