# Supplementary material for: Effects of Wnt5a overexpression in spinal cord injury
Source: J Cell Mol Med. 2021 May 3;25(11):5150–63. doi: 10.1111/jcmm.16507 (PMC8178287; doi:10.1111/jcmm.16507)
Supplement: Supplementary file 5 — Table S4 [file JCMM-25-5150-s006.pdf]

		Rostro-caudal levels (mm from epicenter)																				
		**5.94**	**5.28**	**4.62**	**3.96**	**3.30**	**2.64**	**1.98**	**1.32**	**0.66**	**Epi**	**Epi**	**-0.66**	**-1.32**	**-1.98**	**-2.64**	**-3.30**	**-3.96**	**-4.62**	**-5.28**	**-5.94**	
**GFP**	**Mean**	5.3	5.6	6.9	6.6	5.9	6.9	8.7	8.4	9.2	8.3	10.2	10.3	11.0	10.0	9.8	9.0	9.3	8.5	9.3	8.0	**7 dpi**
	**SEM**	1.7	1.43	1.6	1.3	0.3	0.5	0.9	0.4	0.8	0.6	1.0	1.0	1.1	1.7	0.6	0.8	1.2	0.9	0.3	0.6	
**Wnt5a**	**Mean**	6.1	5.7	5.4	5.7	5.9	6.2	6.7	8.3	8.1	6.4	7.7	7.8	8.5	9.2	10.7	8.7	9.2	8.1	9.4	10.1	
	**SEM**	1.0	1.3	0.8	0.6	0.4	0.7	1.0	0.9	2.0	1.0	0.8	0.4	0.4	0.6	1.6	1.0	1.1	0.9	1.6	1.2	
**GFP**	**Mean**	8.8	9.2	8.7	10.1	10.5	9.3	9.7	11.3	13.2	13.7	11.2	11.7	12.6	11.3	10.3	10.1	10.4	8.8	8.6	9.5	**14 dpi**
	**SEM**	1.3	0.9	1.5	1.7	1.6	0.4	0.4	0.6	1.0	1.5	1.3	0.8	1.0	0.7	1.4	1.0	0.3	0.5	0.2	0.4	
**Wnt5a**	**Mean**	7.3	7.9	6.9	7.4	9.7	8.0	8.8	11.7	13.5	12.6	10.2	13.8	13.4	12.4	11.0	10.6	8.6	8.1	9.0	10.2	
	**SEM**	1.1	1.9	0.6	0.8	0.7	1.7	1.5	1.5	1.3	0.9	0.6	0.7	1.6	0.5	1.0	2.0	1.1	1.5	1.2	1.4	

Table S4. Table showing data obtained from the densitometric analysis of the astroglial response at 7 and 14 days post-injury (dpi). Please note that data obtained from the evaluation of this parameter at 126 dpi can be found in Figure 5. Data represent the percentage of glial fibrillary acidic protein (GFAP)+ area vs. total spinal cord area in each analyzed rostrocaudal level, and are presented as mean ± SEM. GFP group, lesioned animals injected with a lentiviral vector generated to overexpress GFP; Wnt5a group, lesioned animals injected with a lentiviral vector generated to overexpress both GFP and Wnt5a.
