# Supplementary material for: Effects of Wnt5a overexpression in spinal cord injury
Source: J Cell Mol Med. 2021 May 3;25(11):5150–63. doi: 10.1111/jcmm.16507 (PMC8178287; doi:10.1111/jcmm.16507)
Supplement: Supplementary file 6 — Table S5 [file JCMM-25-5150-s001.pdf]

		Rostro-caudal levels (mm from epicenter)																				
		**5.94**	**5.28**	**4.62**	**3.96**	**3.30**	**2.64**	**1.98**	**1.32**	**0.66**	**Epi**	**Epi**	**-0.66**	**-1.32**	**-1.98**	**-2.64**	**-3.30**	**-3.96**	**-4.62**	**-5.28**	**-5.94**	
**GFP**	**Mean**	3.8	3.63	4.3	5.0	6.7	10.2	12.4	15.6	20.9	29.6	31.9	28.5	21.6	17.2	14.8	11.7	12.3	10.5	9.5	9.2	**7 dpi**
	**SEM**	0.4	0.6	0.5	0.3	0.5	1.6	2.3	2.1	2.6	2.2	1.7	0.6	1.8	1.1	0.3	1.2	0.7	0.5	0.6	0.5	
**Wnt5a**	**Mean**	7.27	6.4	6.6	6.4	7.1	7.5	9.5	14.5	23.4	30.3	31.8	30.7	21.5	17.7	15.8	12.7	10.7	11.0	9.2	11.4	
	**SEM**	1.4	1.6	0.7	0.2	0.6	0.4	1.2	1.0	1.9	1.2	0.8	1.1	1.6	1.5	1.8	0.9	1.1	1.3	0.8	1.2	
**GFP**	**Mean**	5.9	5.7	6.7	6.5	8.3	7.4	10.6	15.3	19.6	29.6	30.9	24.3	18.9	13.8	11.6	10.6	10.8	9.6	9.8	8.7	**14 dpi**
	**SEM**	0.4	0.5	0.3	0.4	1.5	0.3	1.5	1.9	2.8	1.4	1.9	1.1	1.6	1.3	1	0.4	0.8	0.6	1.0	0.9	
**Wnt5a**	**Mean**	7.0	7.8	7.2	6.1	7.1	9.5	10.6	13.6	19.2	30.4	29.9	26.4	17.9	12.6	12.6	12.8	11.1	10.3	11.9	11.6	
	**SEM**	1.2	0.9	0.8	1.3	2.9	2.3	1.5	1.5	1.3	2.7	1.2	1.5	1.4	1.0	2.4	1.5	4.4	2.7	2.0	1.1	

Table S5. Table showing data obtained from the densitometric analysis of the microglia/macrophage cell response at 7 and 14 days post-injury (dpi). Please note that data obtained from the evaluation of this parameter at 126 dpi can be found in Figure 6. Data represent the percentage of ionized calcium binding adaptor molecule 1 (Iba1)+ area vs. total spinal cord area in each analyzed rostrocaudal level, and are presented as mean ± SEM. GFP group, lesioned animals injected with a lentiviral vector generated to overexpress GFP; Wnt5a group, lesioned animals injected with a lentiviral vector generated to overexpress both GFP and Wnt5a.
