# Supplementary material for: Effects of Wnt5a overexpression in spinal cord injury
Source: J Cell Mol Med. 2021 May 3;25(11):5150–63. doi: 10.1111/jcmm.16507 (PMC8178287; doi:10.1111/jcmm.16507)
Supplement: Supplementary file 7 — Table S6 [file JCMM-25-5150-s008.pdf]

	Paw positions		Toe clearance		Stepping		Tail position	
	**GFP**	**Wnt5a**	**GFP**	**Wnt5a**	**GFP**	**Wnt5a**	**GFP**	**Wnt5a**
**1 dpi**	0 ± 0	0 ± 0	0 ± 0	0 ± 0	0 ± 0	0 ± 0	0 ± 0	0 ± 0
**3 dpi**	0 ± 0	0 ± 0	0 ± 0	0 ± 0	0 ± 0	0 ± 0	0 ± 0	0 ± 0
**7 dpi**	0 ± 0	0 ± 0	0 ± 0	0 ± 0	0 ± 0	0 ± 0	0 ± 0	0 ± 0
**14 dpi**	0 ± 0	0.07 ± 0.07	0 ± 0	0 ± 0	2.07 ± 0.53	1.14 ± 0.44	0 ± 0	0 ± 0
**21 dpi**	0.4 ± 0.16	0.8 ± 0.29	0 ± 0	0 ± 0	3.1 ± 0.77	2.9 ± 0.64	0.2 ± 0.13	0.1 ± 0.1
**28 dpi**	0.7 ± 0.33	0.3 ± 0.15	0.3 ± 0.3	0 ± 0	3.4 ± 0.81	3.5 ± 0.86	0.3 ± 0.15	0.4 ± 0.16
**42 dpi**	0.56 ± 0.29	0.3 ± 0.21	0.44 ± 0.33	0 ± 0	3.78 ± 0.86	4 ± 0.79	0.56 ± 0.18	0.50 ± 0.17
**56 dpi**	0.56 ± 0.29	0.4 ± 0.22	0.56 ± 0.33	0 ± 0	3.89 ± 0.89	3.4 ± 0.85	0.56 ± 0.18	0.50 ± 0.17
**70 dpi**	0.44 ± 0.24	0.4 ± 0.22	0.33 ± 0.23	0 ± 0	3.89 ± 0.89	3.5 ± 0.86	0.44 ± 0.18	0.50 ± 0.17
**84 dpi**	0.44 ± 0.24	0.4 ± 0.22	0.33 ± 0.23	0 ± 0	3.44 ± 0.84	3.2 ± 0.9	0.44 ± 0.18	0.50 ± 0.17
**98 dpi**	0.38 ± 0.18	0.3 ± 0.15	0.63 ± 0.42	0 ± 0	2.75 ± 1	2.4 ± 0.98	0.5 ± 0.19	0.4 ± 0.16
**112 dpi**	0.38 ± 0.18	0.2 ± 0.13	0.75 ± 0.52	0 ± 0	2.75 ± 1	2.4 ± 0.98	0.5 ± 0.19	0.4 ± 0.16
**126 dpi**	0.38 ± 0.18	0.2 ± 0.13	0.63 ± 0.42	0 ± 0	2.38 ± 1	2.4 ± 0.98	0.5 ± 0.19	0.4 ± 0.16

Table S6. Table showing data obtained from the separate analysis of the following individual aspects of locomotion evaluated in the 21-point Basso, Beattie and Bresnahan (BBB) open-field test: paw positions, toe clearance, stepping and tail positions. Please note that data obtained from the evaluation of coordination can be found in Figure 7. Analysis was performed at 1, 3, 7, 14, 21, 28, 42, 56, 70, 84, 98, 112 and 126 days post-injury (dpi). Data are presented as mean ± SEM. GFP group, lesioned animals injected with a lentiviral vector generated to overexpress GFP; Wnt5a group, lesioned animals injected with a lentiviral vector generated to overexpress both GFP and Wnt5a.
