# Supplementary material for: Effects of Wnt5a overexpression in spinal cord injury
Source: J Cell Mol Med. 2021 May 3;25(11):5150–63. doi: 10.1111/jcmm.16507 (PMC8178287; doi:10.1111/jcmm.16507)
Supplement: Supplementary file 8 — Table S7 [file JCMM-25-5150-s005.pdf]

		Pre-injury	105 dpi	126 dpi
**Base of support (mm)**	**GFP**	32.49 ± 0.9	50.71 ± 1.43***	52.22 ± 0.84***
	**Wnt5a**		56.81 ± 2.51***	52.06 ± 3.17***
**Stride length (mm)**	**GFP**	121.98 ± 1.67	115.71 ± 0.39	111.69 ± 2.47
	**Wnt5a**		124.16 ± 4.59	122.24 ± 2.93
**Duty cycle (%)**	**GFP**	63.21 ± 0.58	78.06 ± 2.7***	79.93 ± 2.53***
	**Wnt5a**		74.36 ± 2.41***	77.64 ± 0.78***
**Swing duration (s)**	**GFP**	0.127 ± 0.003	0.077 ± 0.005***	0.071 ± 0.006***
	**Wnt5a**		0.093 ± 0.005**	0.087 ± 0.005***
**Swing speed (m/s)**	**GFP**	0.975 ± 0.019	1.59 ± 0.09***	1.76 ± 0.14***
	**Wnt5a**		1.48 ± 0.05***	1.6 ± 0.08***
**Stand duration (s)**	**GFP**	0.21 ± 0.005	0.28 ± 0.015**	0.29 ± 0.02**
	**Wnt5a**		0.27 ± 0.016**	0.27 ± 0.02**

Table S7. Table showing data obtained from the analysis of the following gait parameters evaluated using the CatWalk gait analysis system: base of support, stride length, duty cycle, swing duration, swing speed and stand duration. Please note that data obtained from the evaluation of regularity index, frequency of AB step pattern and print positions can be found in Figure 7. Analysis was performed both before (to obtain pre-injury values) and after injury at 105 and 126 days post-injury (dpi). Data are presented as mean ± SEM. **, $p < 0.01$ and *** $p < 0.001$ vs pre-injury. GFP group, lesioned animals injected with a lentiviral vector generated to overexpress GFP; Wnt5a group, lesioned animals injected with a lentiviral vector generated to overexpress both GFP and Wnt5a.
